# Forskolin-Induced Lipid-Droplet Remodeling Is Associated with Improved Redox Homeostasis and Cryotolerance of Vitrified Porcine Oocytes and Parthenogenetic Embryos

**DOI:** 10.3390/cells15201909

**Published:** 2026-10-20

**Authors:** Jiehuan Xu, Yongliang Chen, Shaoli Cai, Jun Gao, Mengqian He, Caifeng Wu, Jianjun Dai, Shushan Zhang, Yi Bian, Lingwei Sun

**Affiliations:** 1 Shanghai Municipal Key Laboratory of Agri-Genetics and Breeding, Shanghai Engineering Research Center of Breeding Pig, Institute of Animal Science and Veterinary Medicine, Shanghai Academy of Agricultural Sciences, Shanghai 201106, China; jiehuanxu@saas.sh.cn (J.X.); gaojun@saas.sh.cn (J.G.); he1037247863@163.com (M.H.); wucaifengwcf@163.com (C.W.); daijianjun@saas.sh.cn (J.D.); smalltreexj@126.com (S.Z.); 2 Key Laboratory of Livestock and Poultry Resources Evaluation and Utilization, Ministry of Agriculture and Rural Affairs, Shanghai 201106, China; 3 Jiading District Meishan Pig Breeding Center, Shanghai 201807, China; yardlychen@163.com; 4 Shanghai Jiading District Animal Disease Prevention and Control Center, Shanghai 201899, China; 5 College of Animal Sciences, Guizhou University, Guiyang 550025, China; caishaoliwww@163.com

**Keywords:** porcine oocytes, parthenogenetic embryos, forskolin, lipolysis, lipid droplets, oxidative stress, cryopreservation

## Abstract

Cryopreservation of mammalian oocytes and embryos is essential for the long-term conservation of livestock germplasm resources. Porcine oocytes and embryos accumulate high levels of intracellular lipids. This heavy lipid load aggravates vitrification-related oxidative stress and mitochondrial redox disturbance, greatly reducing cellular cryotolerance and hindering reliable preservation of indigenous pig germplasm. This study aimed to develop a non-invasive metabolic intervention strategy to mitigate lipid-associated oxidative cryodamage and improve the post-thaw quality of porcine oocytes and parthenogenetic embryos. Porcine cumulus–oocyte complexes were treated with forskolin, an adenylate cyclase activator, throughout in vitro maturation. For embryo assays, parthenogenetic embryos received continuous forskolin supplementation during oocyte maturation and were vitrified at Day 2 (cleavage stage). Vitrification disrupted lipid-droplet structural integrity and perturbed multiple metabolic pathways. Enrichment of starch, sucrose and galactose metabolism should be interpreted with caution as computational outputs, alongside changes in glycine, serine and threonine metabolism. Cryoinjury further induced mitochondrial depolarization, excessive reactive oxygen species production, redox imbalance and early apoptosis, ultimately impairing cell viability and embryonic developmental competence. Continuous forskolin exposure during oocyte in vitro maturation (with matching treatment for embryo groups) triggered lipid-droplet remodeling. This remodeling was associated with restored mitochondrial redox homeostasis and suppressed oxidative stress and apoptosis in warmed oocytes and embryos. Forskolin treatment enhanced post-thaw survival and cleavage rates of vitrified oocytes, as well as survival of cryopreserved embryos. By contrast, blastocyst formation rate showed only a numerical, non-significant increase. In conclusion, non-invasive forskolin-driven lipid-droplet remodeling can mitigate vitrification-triggered oxidative damage to lipid droplets and mitochondria. This work provides a practical pre-cryopreservation optimization strategy based on redox protection. It supports improved oocyte experimental protocols and offers useful references for germplasm conservation of rare and endangered pig breeds.

## 1. Introduction

Pigs boast abundant genetic resources, yet numerous indigenous pig breeds face a growing risk of genetic diversity decline and even extinction. Especially given the ongoing threat of African swine fever infection [1], long-term cryopreservation of gametes and embryos has grown far more critical. This biobanking strategy serves as a fundamental safeguard to preserve valuable pig germplasm. Vitrification is a standardized, repeatable and reliable technique widely used for livestock germplasm conservation as well as human assisted reproduction [2]. Since the first successful vitrification cryopreservation of mouse oocytes was reported in 1989 [3], this technology has been adopted for long-term oocyte and embryo banking across humans and farm animals. Up to now, viable cryopreservation via vitrification has been documented in more than 20 species, including rats, goats, sheep, cattle and rabbits [4].

Unfortunately, vitrification remains imperfect for porcine oocytes and embryos. Cryoinjury impairs cellular physiological function, making it hard to use this method for stable long-term preservation of elite breeding sows [5]. Globally, live piglets derived from vitrified porcine embryos are only sporadically reported, and this approach cannot support large-scale commercial breeding. Unlike oocytes from model animals such as mice, porcine oocytes are highly susceptible to low-temperature damage [6]. Oocytes incubated at 15 °C for just 15 min nearly lose all viability [7]. Healthy live offspring were only obtained from vitrified-warmed GV-stage porcine oocytes in 2014 [8] and MII-stage oocytes in 2015 [9].

Poor cryosurvival of porcine oocytes and embryos is commonly attributed to their exceptionally high intracellular lipid level. Excessive lipid accumulation is an inherent, species-specific feature of pig gametes and embryos. Quantitatively, porcine oocytes contain 161 μg lipids per 1000 oocytes. This value is 1.8-fold higher than sheep oocytes, 2.6-fold higher than bovine oocytes, and 6.8-fold higher than mouse oocytes. Most accumulated lipids exist in the form of triglycerides stored in lipid droplets (LDs) [10]. Within oocytes and embryos, LDs participate in many essential biological activities. These processes include embryonic diapause control, oxidative phosphorylation, glycolysis, gluconeogenesis and the tricarboxylic acid cycle [11]. Even so, LDs are not strictly indispensable for embryonic development. Previous studies have confirmed that mammalian embryos can develop normally after partial LD removal [12].

During vitrification, abundant intracellular lipids hinder full water replacement by cryoprotective agents. Permeation of cryoprotectants becomes insufficient in porcine oocytes and embryos. Ice crystals easily form inside cells during cooling or warming, triggering mechanical damage to multiple subcellular organelles [13]. Excess lipids also slow down vitrification penetration. Cells require longer exposure to vitrification solutions, raising risks of chemical cytotoxicity. Meanwhile, the procedure demands stricter operation precision with little room for human error.

Traditional delipidation mainly relies on oocyte centrifugation combined with micromanipulation. This physical strategy improves post-thaw survival of porcine oocytes [14], but brings notable drawbacks. Centrifugation and micromanipulation disrupt organelle distribution and damage the zona pellucida. These injuries increase risks of pathogen contamination [15] and permanent physical cellular harm. Manual micromanipulation calls for experienced technicians and costly equipment, restricting high-throughput cryopreservation of oocytes and embryos. Furthermore, centrifugal delipidation disturbs LD distribution, breaks cumulus–oocyte complex integrity and disrupts cellular energy supply. These changes interfere with oocyte meiosis [16] and reduce subsequent embryonic developmental capacity [17]. Bulk lipid removal via physical methods ultimately impairs oocyte developmental potential. Therefore, there is an urgent need to develop non-invasive protocols to lower cellular lipid content and strengthen cryoresistance in porcine oocytes and embryos.

Multiple natural compounds can modulate cellular lipid metabolism and reduce lipid deposition [18]. These findings inspired us to explore non-invasive metabolic lipid-lowering for porcine reproductive cells. Forskolin is a natural active ingredient extracted from Coleus forskohlii. It accelerates lipolysis and regulates overall cellular metabolism and physiological activities [19,20]. As a classic cyclic adenosine monophosphate (cAMP) modulator, forskolin mediates cAMP-dependent signaling. cAMP signaling supplies energy to support mammalian oocyte meiotic arrest, fertilization, and embryonic development [21]. Importantly, due to their high lipid content and cryosensitive phenotype, porcine oocytes serve as a well-established translational model for human oocyte cryobiology, making our cAMP-mediated lipid regulation findings broadly informative for mammalian reproductive cryopreservation and clinical assisted reproduction.

On these grounds, the present study aimed to clarify whether forskolin-induced lipolysis could relieve vitrification damage and improve post-thaw function of porcine oocytes and embryos. We added forskolin to porcine oocyte IVM medium, as well as the subsequent PZM-3 embryo culture medium. We systematically detected how forskolin affected LD morphology and lipid metabolism. We also evaluated mitochondrial function, cellular redox balance, early apoptosis, post-thaw survival rate and embryonic developmental competence after vitrification.

## 2. Materials and Methods

### 2.1. Chemicals

Medium-199 with HEPES and fetal bovine serum (FBS) were purchased from GIBCO, USA. Human chorionic gonadotropin and pregnant mare serum gonadotropin were purchased from Ningbo third hormone products Co, China (Ningbo, China). Nile red and fluorescein diacetate (FDA) were purchased from Shanghai Yuanye Biotechnology Co., China (Shanghai, China). 2′,7′-Dichlorodihydrofluorescein diacetate (DCFH-DA), 5,5′,6,6′-tetrachloro-1,1′,3,3′-tetraethyl-imidacarbocyanine iodide (JC-1) and Annexin-V were purchased from Beyotime Biotech. Inc., China (Shanghai, China). Other reagents were purchased from Sigma-Aldrich, USA. Forskolin (F6886, Sigma-Aldrich, St. Louis, MO, USA; purity ≥ 98% by HPLC) was dissolved in dimethyl sulfoxide (DMSO) to prepare a 10 mM stock solution and stored at −20 °C. Owing to its hydrophobicity, the stock was diluted with the in vitro maturation (IVM) medium to a final working concentration of 10 μM, with the final DMSO concentration adjusted to 0.1% (*v*/*v*). An equal volume of DMSO alone was added to the IVM medium of the control group to match the vehicle exposure.

### 2.2. Experimental Treatments

#### 2.2.1. Oocyte IVM and Oocyte-Related Assessments

Four experimental groups were designed for oocyte experiments in this study. The first group consisted of fresh oocytes obtained after standard IVM. The second group included oocytes continuously treated with 10 μM forskolin during the entire in vitro maturation process. The third group was composed of untreated mature oocytes that underwent routine vitrification and warming procedures. The fourth group was subjected to 10 μM forskolin supplementation during in vitro maturation followed by identical vitrification and warming treatments.

#### 2.2.2. Vitrification–Thawing, Parthenogenetic Activation and Embryo Developmental Evaluation

Following IVM, oocytes were assigned to four treatment groups: Fresh, Forskolin, Vitrified, and Forskolin + Vitrified. Oocytes in the Fresh group were collected immediately after IVM without vitrification. The Forskolin group received forskolin supplementation during IVM but was not subjected to vitrification. Oocytes in the Vitrified group underwent vitrification–warming without forskolin supplementation. For oocyte experiments, forskolin was added continuously for the entire 44 h of oocyte IVM. Vitrification was performed on MII-stage oocytes after completion of 44 h in vitro maturation. For the Forskolin + Vitrified group, oocytes were cultured with forskolin throughout IVM before vitrification–thawing. For embryo experiments, parthenogenetic embryos were vitrified at Day 2 post activation (cleavage stage). In embryo-related groups receiving forskolin, continuous forskolin exposure was maintained throughout both oocyte IVM and subsequent embryo in vitro culture up to the time of vitrification, rather than a single pre-vitrification pretreatment. Post-warming embryo survival was assessed 2 h after thawing; embryos with intact morphology and clear membrane integrity were defined as surviving embryos.

### 2.3. Oocytes IVM

Ovaries were collected from prepubertal gilts at a local commercial slaughterhouse. Ovaries were obtained from already slaughtered prepubertal gilts and transported in 0.9% (*w*/*v*) saline at 39 °C within 1 h. Cumulus–oocyte complexes (COCs) with porcine follicular fluid were aspirated from 2 to 6 mm follicles using a 10 mL syringe. COCs were washed three times with in vitro maturation medium consisting of Medium-199 supplemented with 10% FBS, 10% porcine follicular fluid, 10 IU/mL human chorionic gonadotropin, 10 IU/mL pregnant mare serum gonadotropin, 0.57 mM cysteine, 75 μg/mL penicillin, 50 μg/mL streptomycin, and 10 ng/mL epidermal growth factor. The concentration of forskolin referred to Prates’ study [22]. A total of 60 COCs were matured for 44 h in 500 μL of maturation medium, containing 10 μM forskolin or not, at 39 °C in a humidified atmosphere of 5% CO_2._ After 44 h culture, COCs were transferred into 0.1% hyaluronidase to obtain cumulus-denuded oocytes, transferred into Medium-199 and observed under a microscope.

For the maturation assay, 5 independent biological replicates were performed (*n* = 5), with 150 oocytes per group in each replicate. Each biological replicate consisted of three technical replicates of 50 oocytes. A total of 750 oocytes per group were analyzed. Those oocytes with a visible first polar body were considered as mature (MII stage) oocytes.

### 2.4. Parthenogenetic Activation (PA) and In Vitro Embryo Culture (IVC)

PA was performed to produce early embryos. To assess embryonic developmental competence, the cleavage rate was evaluated on Day 2 after parthenogenetic activation, and the blastocyst formation rate was assessed on Day 6. Five independent biological replicates were performed (*n* = 5). In each biological replicate, 150 oocytes were included per group and randomly divided into three technical replicates of 50 oocytes each. Thus, a total of 750 oocytes were analyzed per group across the five independent biological replicates. The composition of the electric activation medium and the experimental procedures were described by Li [23]. Briefly, ten oocytes were transferred into a 0.5 mm gap chamber filled with electric activation medium connected to an Electro Cell Manipulator (BTX 2001, Genetronics, San Diego, CA, USA). Oocytes were subjected to an electrical pulse of 60 V for 60 μs. Activated oocytes were cultured in porcine zygote medium-3 (PZM-3) [24] at 39 °C under humidified atmosphere with 5% CO_2_ for 6 days. For the forskolin-treated embryo group, 10 μM forskolin was added to both IVM medium and PZM-3, maintaining continuous exposure from the start of oocyte maturation until embryo vitrification, to continuously decrease intracellular lipid content.

### 2.5. Vitrification and Warming

Each ten porcine oocytes or embryos were equilibrated in equilibrium solution (Medium-199 supplemented with 7.5% ethylene glycol, 7.5% dimethyl sulphoxide, and 20% FBS) for 3 min, transferred into vitrification solution (Medium-199 supplemented with 15% ethylene glycol, 15% dimethyl sulphoxide, 20% FBS and 0.4 M sucrose) for 20 s, loaded onto a Cryotop^®^, immediately immersed in liquid nitrogen, and stored for at least one month. The time for placing the oocytes or embryos into the vitrification solution to immersion in liquid nitrogen should be controlled to not exceed 1 min. During warming, the cryopreserved oocytes or embryos were successively immersed in Medium-199 (for oocyte warming) or PZM-3 (for embryo warming) containing 20% FBS and 0.3 M or 0.15 M sucrose at 39 °C in a humidified atmosphere of 5% CO_2_ for 5 min each, and then recovered in Medium-199 or PZM-3 with 10% FBS for an hour.

### 2.6. Ultrastructural Observation

Oocytes or embryos were fixed in 2.5% glutaraldehyde at 4 °C for 2 h and washed twice with pre-cooled PBS for 10 min, embedded in 4% agar, fixed with 1% osmium tetroxide at 4 °C for 1 h, washed three times with PBS, dehydrated in a graded ethanol series, and then washed with propylene oxide twice for 10 min each. Then, oocytes or embryos were soaked with EPON 812, embedded in an oven at 60 °C for 48 h, sectioned, stained with lead citrate electron, and dried naturally. After that, they were observed and photographed under a transmission electron microscope (H-7650, Hitachi, Tokyo, Japan).

### 2.7. Quantification of Lipid Droplet Content

In each independent biological replicate, three individual oocytes or embryos per group were analyzed, and the measurements obtained from these three individuals were averaged to generate one replicate-level value. Thirty independent biological replicates were performed (*n* = 30), resulting in a total of 90 oocytes or embryos analyzed per group. Oocytes or embryos were stained with 10 μM Nile red at 39 °C for 15 min, washed 3 times with PBS, and observed using a laser-scanning confocal microscope (LSM980, Zeiss, Oberkochen, Germany). The quantities and areas of maximum cross-section of LDs in oocytes or embryos were determined and statistically analyzed using Image J software 1.52a (NIH, Bethesda, MD, USA). One independent biological replicate corresponds to one independent ovary collection and an independent full IVM experimental run.

### 2.8. Metabolomics Analysis

For metabolomics analysis, six biological replicates were set for each group. Every 100 oocytes were pooled as one biological sample and transferred into 2 mL of serum-free Medium 199, followed by incubation at 39 °C for 12 h. This additional 12 h serum-free incubation was designed to eliminate exogenous lipid and metabolite carry-over derived from serum supplements in the in vitro maturation medium to avoid interference with untargeted metabolomic measurements. Importantly, all collected oocyte samples (Fresh, Forskolin, and Vitrified groups) received this identical 12 h serum-free incubation under matched culture conditions to exclude group-specific bias. We acknowledge that prolonged serum-free incubation may alter intrinsic oocyte metabolic profiles, which represents a potential confounding factor for data interpretation.

Oocytes were manually selected under a microscope, discarded from Medium 199, transferred to cryovials, snap-frozen in liquid nitrogen and stored at −80 °C. Samples were subjected to five repeated freeze–thaw cycles (thawed at 4 °C and refrozen at −80 °C). One milliliter of 75% methanol was added for metabolite extraction; samples were sonicated on ice for 15 min and centrifuged at 13,000 r/min for 10 min at 4 °C. The supernatant was collected, and the precipitate was re-extracted with another 1 mL of 75% methanol followed by 15 min sonication. The two supernatants from the same sample were combined and freeze-dried. The dried extract was reconstituted in 60 μL of 1% formic acid solution, centrifuged at 13,000 r/min for 10 min at 4 °C, and the supernatant was filtered through a 0.22 μm filter for subsequent LC-MS analysis.

Quality control (QC) samples were generated by mixing equal volumes of extracts from all experimental samples. Three pooled QC samples were injected throughout the LC-MS run sequence to evaluate instrument stability, retention-time consistency and peak-intensity drift. Metabolic features with coefficient of variation (CV) > 30% across QC replicates were excluded from downstream statistical analysis.

Liquid chromatography–mass spectrometry detection was performed using a Dionex U3000 UHPLC system (Thermo Fisher Scientific, Waltham, MA, USA) coupled to a QE Plus high-resolution mass spectrometer (Thermo Fisher Scientific, USA). Raw MS data were acquired using Xcalibur 4.3 (Thermo Fisher Scientific, USA).

Raw data underwent sequential pre-processing, including missing-value filtering, missing-value imputation, data normalization, QC verification, data conversion, and log-transformation. Multivariate statistical analysis was performed in R software (R-4.2.1, R Foundation for Statistical Computing, Vienna, Austria) using the ropls package (v1.34.1) to perform differential metabolite statistics and OPLS-DA modeling. Metabolite cluster analysis and VIP calculation were implemented in Python 3.9 using the SciPy library (v1.10.1). Differential metabolites were screened according to the following criteria: variable importance in projection (VIP) ≥ 1.0, |log2(fold-change)| ≥ 0.585 (corresponding to fold-change ≥ 1.5 or ≤0.67), and adjusted-P < 0.05. The Benjamini–Hochberg procedure was implemented for multiple-testing correction to control the false-discovery rate (FDR).

Metabolite identification confidence was reported following Metabolomics Standards Initiative (MSI) guidelines. Metabolite annotation was performed by matching accurate *m*/*z* value and retention-time information against the Human Metabolome Database (HMDB, version 5.0), without MS/MS spectral confirmation; therefore, annotations correspond to MSI Level-3 confidence All metabolite assignments including okadaic acid, graveolinine and 9-Oxoasimicinone are considered putative identifications and require further verification via reference standards. Only features with mass error less than 5 ppm were retained.

Annotated metabolites were classified based on information retrieved from the HMDB database (version 5.0). The Kyoto Encyclopedia of Genes and Genomes (KEGG, release 108.0) database together with Fisher’s exact test were used for KEGG pathway enrichment analysis. Pathways with *p* < 0.05 were considered significantly enriched in the metabolite set.

Metabolic features were detected in both positive and negative electrospray ionization modes. The complete untargeted metabolomic dataset, comprising the normalized peak intensities of all detected features across the three experimental groups (Fresh, Forskolin, and Vitrified; six biological replicates each) together with three pooled QC samples, is provided in Supplementary Table S1.

### 2.9. Determination of Mitochondrial Membrane Potential

In each independent biological replicate, three individual oocytes or embryos per group were analyzed, and the measurements obtained from these three individuals were averaged to generate one replicate-level value. Thirty independent biological replicates were performed (*n* = 30), resulting in a total of 90 oocytes or embryos analyzed per group. The mitochondrial membrane potential (MMP) was detected with a JC-1 detection kit. Oocytes or embryos were incubated with 2 μM JC-1 at 39 °C for 20 min, washed 3 times with PBS and observed with a laser-scanning confocal microscope. Poly-JC-1 was monitored in red fluorescence when MMP was high, while mono-JC-1 was monitored in green fluorescence when MMP was low. The ratio of red fluorescence intensity (JC-1 polymer)/green fluorescence intensity (JC-1 monomer) was the value of MMP. One independent biological replicate corresponds to one independent ovary collection and an independent full IVM experimental run.

### 2.10. Determination of ROS Generation

Five independent biological replicates were performed. In each biological replicate, 30 oocytes or embryos were included per group and randomly divided into three technical replicates, with 10 oocytes or embryos in each technical replicate. The level of ROS generation was detected by DCFH-DA. Oocytes or embryos were stained with 10 μM DCFH-DA at 39 °C for 20 min, washed three times with PBS, and observed by a fluorescence microscope. The fluorescence intensity in each oocyte or embryo represented its level of ROS generation.

### 2.11. Determination of Apoptosis

Early apoptosis was detected by Annexin V-mCherry. Five independent biological replicates were performed. In each biological replicate, 90 oocytes or embryos were included per group and randomly divided into three technical replicates, with 30 oocytes or embryos in each technical replicate. Oocytes or embryos were washed three times with DPBS and transferred into 195 μL Annexin V-FITC binding buffer containing 5 μL Annexin V-mCherry for 20 min at room temperature in the dark, washed three times with DPBS and observed with a fluorescence microscope.

### 2.12. Determination of Viability

Fluorescein diacetate (FDA) was used to determine viability. Five independent biological replicates were performed. In each biological replicate, 90 oocytes or embryos were included per group and randomly divided into three technical replicates, with 30 oocytes or embryos in each technical replicate. Oocytes or embryos were incubated with 5 μg/mL FDA at 39 °C for 10 min, washed three times with PBS, and observed with a fluorescence microscope. Those with green fluorescence were viable.

### 2.13. Statistical Analysis

Independent biological replicates were considered the experimental units for statistical analysis. Statistical analyses were performed using SPSS 17.0 (SPSS Inc., Chicago, IL, USA). Comparisons between two groups were performed using an independent-samples *t*-test, whereas comparisons among more than two groups were analyzed using one-way ANOVA followed by LSD multiple-comparison tests. The results were expressed as mean ± standard error of the mean (SEM). A *p* value < 0.05 was considered statistically significant.

## 3. Results

### 3.1. Forskolin Treatment Achieved Comparable Oocyte Maturation

**Figure 1:**
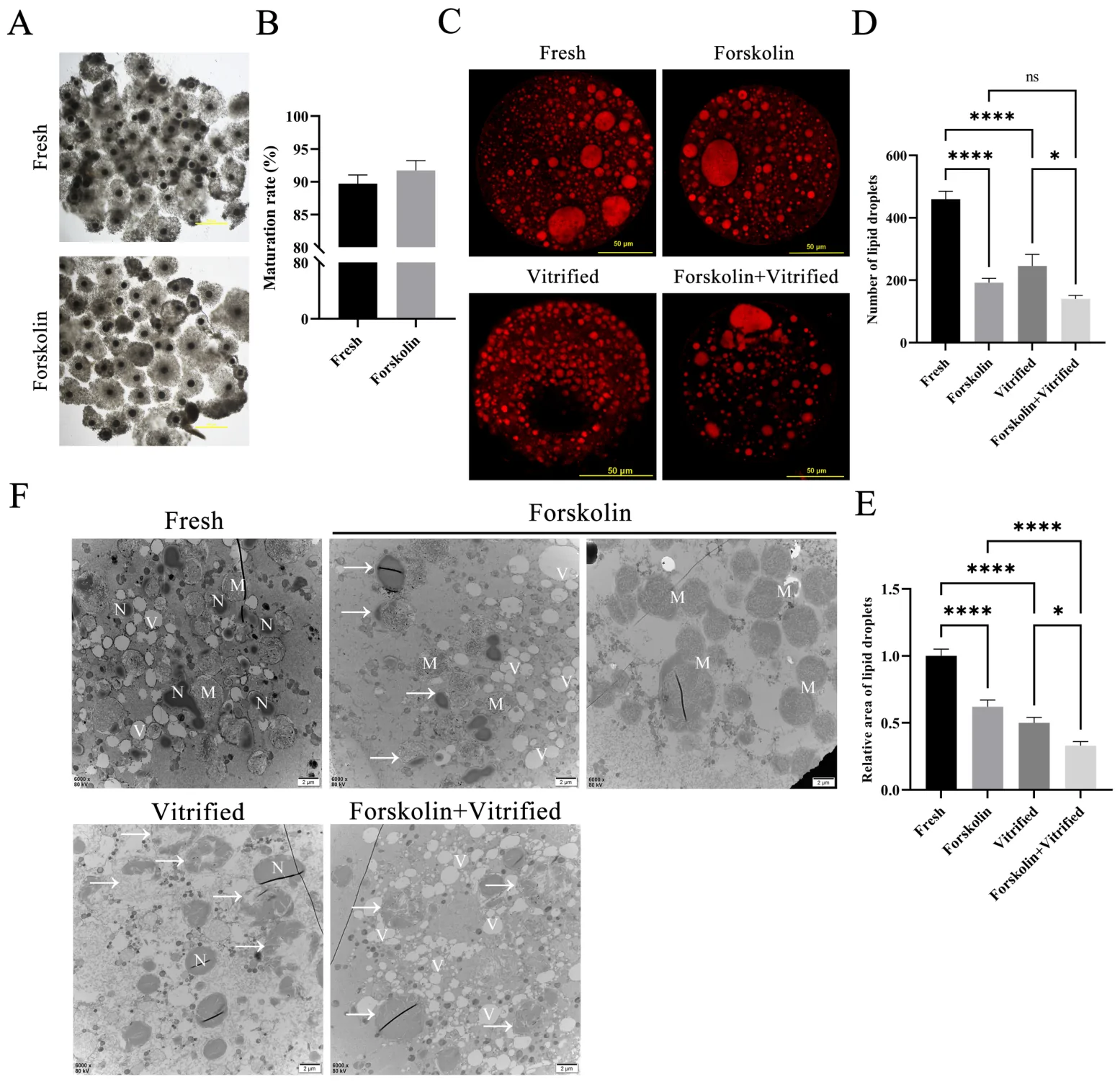
Effects of forskolin treatment before vitrification on porcine oocytes. (**A**) The spread of cumulus cells after oocyte IVM. Scale bar = 500 μm. (**B**) Statistical analysis of oocyte maturation rate (*n* = 5). (**C**) Fluorescent images of LDs stained with Nile red in oocytes. Scale bar = 50 μm. (**D**) Quantitative analysis of the numbers of LDs (*n* = 30). (**E**) Quantitative analysis of the relative areas of LDs (*n* = 30). (**F**). Ultrastructural observation of oocytes. N: Homogeneous LDs; M: Heterogeneous LDs; V: Vacuoles; Arrows: Homogeneous LDs turning into heterogeneous LDs; Scale bar = 2 μm. * *p* < 0.05; **** *p* < 0.0001. “ns”, non-significant (*p* > 0.05).

Cumulus cell support surrounding oocytes is critical for oocyte maturation. We detected cumulus cell expansion and counted oocytes with extruded first polar bodies after IVM to evaluate maturation status. Cumulus expansion levels showed no obvious differences with or without forskolin supplementation following IVM (Figure 1A). First polar body extrusion was used as the marker of oocyte nuclear maturation. Statistical results showed that the oocyte maturation rate of the forskolin group had no significant difference compared with the fresh oocyte control group (*p* > 0.05) (Figure 1B). In short, forskolin does not interfere with regular oocyte maturation.

### 3.2. Forskolin-Associated Lipid-Droplet Remodeling in Oocytes

We performed fluorescence quantitative detection (Figure 1C–E) and ultrastructural observation via transmission electron microscopy (Figure 1F) to analyze lipid droplet (LD) features of oocytes in four groups: fresh control, forskolin-treated, vitrified, forskolin + vitrification. Fresh oocytes contained two types of LDs under TEM: homogeneous LDs and heterogeneous LDs. Homogeneous LDs displayed deep electron staining. Heterogeneous LDs were spherical with transparent linear stripes; heterogeneous LDs were more numerous and larger in size than homogeneous LDs. Dark, round or oval mitochondria distributed evenly around lipid droplets in fresh oocytes.

Compared with fresh control oocytes, forskolin treatment during IVM markedly decreased the total number and relative area of LDs (both *p* < 0.01; Figure 1C–E). TEM results revealed fewer homogeneous LDs in forskolin-treated oocytes. Most residual homogeneous LDs presented partial local heterogenization (white arrows, Figure 1F). These morphological observations suggest that forskolin drives the conversion of homogeneous LDs into heterogeneous LDs and may trigger LD degradation, rather than providing definitive biochemical proof of lipolysis activation.

### 3.3. Vitrification Caused Destruction of LDs in Oocyte and a Lower Lipid Content in Pre-Lipid-Lowering Oocytes

Vitrification and cryopreservation significantly reduced LD number and relative area relative to fresh oocytes (both *p* < 0.01; Figure 1C–E). TEM further explained the ultrastructural changes in lipid loss. After vitrification, most homogeneous LDs were dissolved or internally heterogenized, accompanied by many vacuolar remnants. Heterogeneous LDs became the main residual lipid form after vitrification.

The combination of forskolin treatment and vitrification resulted in the lowest numbers and relative areas of LDs among all groups. When compared with oocytes receiving forskolin treatment alone, vitrification further reduced the relative areas of LDs in forskolin-pretreated oocytes (*p* < 0.01), whereas no significant difference was detected in the numbers of LDs (*p* > 0.05). These data indicate that forskolin-triggered lipolysis reduces basal LD abundance in oocytes before cryopreservation. Although additional LD loss still occurs upon vitrification, qualitative TEM ultrastructural observations indicate that continuous forskolin supplementation remodels LD ultrastructure and may mitigate severe structural disintegration of LDs induced by vitrification; this conclusion is not supported by LD number- and-area quantitative measurements, as the Forskolin + Vitrified group exhibited the lowest LD abundance among all groups.

### 3.4. The Metabolic Differences Between Forskolin-Treatment and Vitrification in Reducing Oocyte Lipid Content

Untargeted metabolomics analysis was conducted to explore potential lipolysis-related mechanisms as well as metabolic differences induced by forskolin exposure and vitrification in porcine oocytes. Only two pairwise comparisons were performed in the present study: forskolin-treated oocytes versus fresh oocytes, and vitrified oocytes versus fresh oocytes. Notably, the Forskolin + Vitrified group was not included in untargeted LC-MS metabolomic profiling; each experimental group contained six biological replicates.

**Figure 2:**
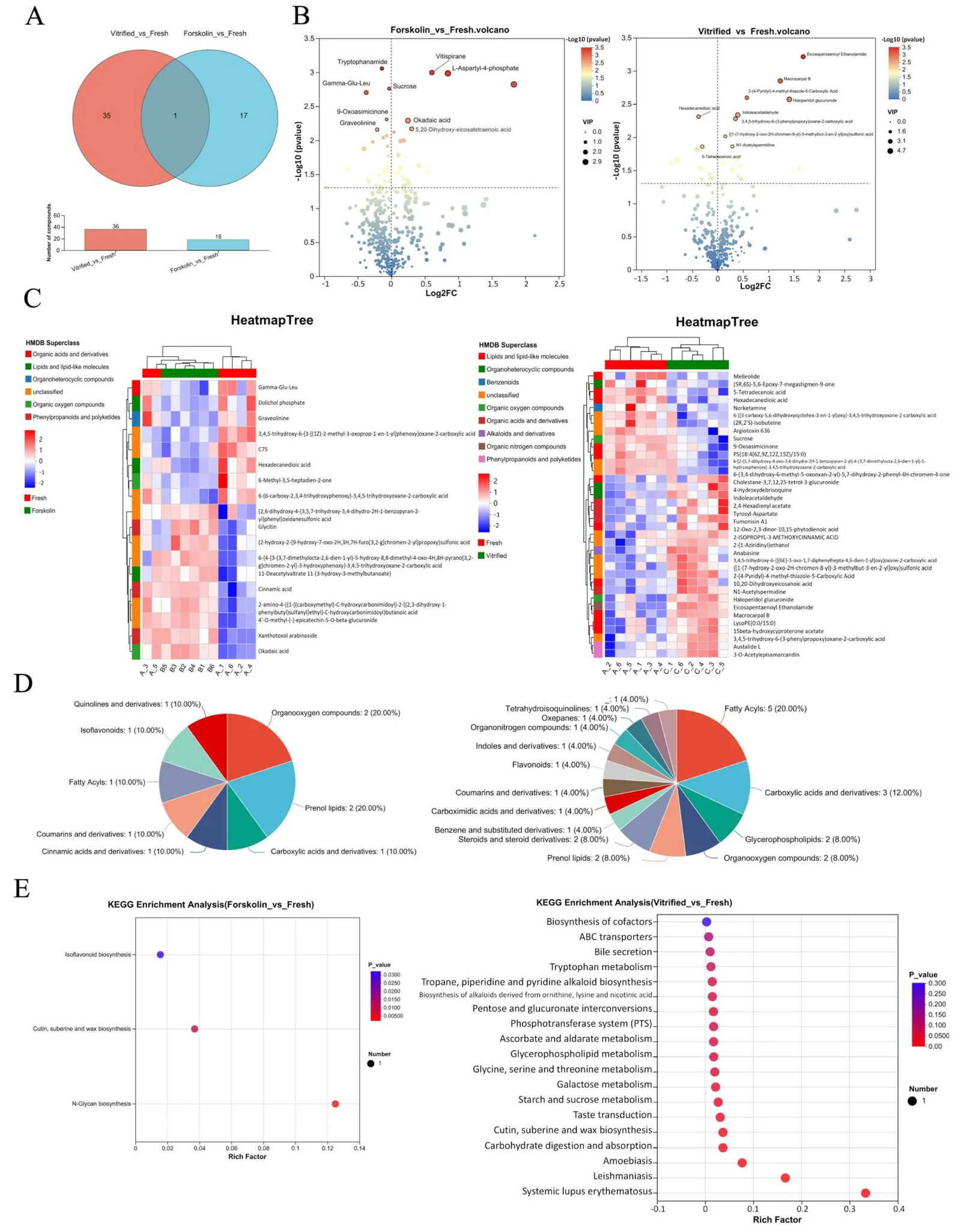
Metabolite profiling in oocytes with forskolin-treatment or underwent vitrification (*n* = 6). (**A**) Venn diagram representing metabolites in common or unique to two metabolic sets. (**B**) Volcano maps were plotted to screen differential metabolites. (**C**) Metabolite clustering analysis with HMDB superclass. Left heatmap tree: Forskolin vs. Fresh; right heatmap tree: Vitrified vs. Fresh. (**D**) HMDB compound classification. Left pie: Forskolin vs. Fresh; right pie: Vitrified vs. Fresh. (**E**) KEGG enrichment analysis. Note: The full output of KEGG enrichment is presented in (**E**), including disease-associated pathways. Disease- and immune-related entries were excluded when we interpreted physiological pathways in the main text.

The Venn diagram identified hexadecanedioic acid (KEGG ID: C19615) as the shared differential metabolite for both comparisons. Among all differential metabolites detected, 17 metabolites were uniquely altered following forskolin treatment, and 35 metabolites specifically changed in vitrified oocytes (Figure 2A). This observation suggests that both forskolin treatment and vitrification may modulate fatty-acid metabolism in oocytes. Nonetheless, the two treatments exhibited distinct metabolic profiles.

Volcano plots visualized all significantly changed metabolites (Figure 2B). In forskolin-treated oocytes, 18 metabolites were upregulated and 9 were downregulated. These metabolites covered lipid peroxidation products (9-Oxoasimicinone), saccharides (sucrose), amino acids and derivatives (tryptophanamide), polypeptides (Gamma-Glu-Leu), alkaloids (graveolinine), organic acids (okadaic acid), phosphate compounds (L-Aspartyl-4-phosphate), and other molecules. Vitrification induced 16 upregulated and 10 downregulated metabolites, which were mostly ketones, aldehydes and organic acids.

We clustered differential metabolites to observe overall expression trends across groups. Heatmap clustering results showed favorable grouping of samples: forskolin group samples (B1–B6) and vitrification group samples (C1–C6) clustered independently (Figure 2C). Metabolites altered by forskolin were classified into multiple HMDB superclasses: organic acids and derivatives, lipids and lipid-like molecules, organoheterocyclic compounds, organic oxygen compounds, phenylpropanoids and polyketides. Metabolites changed by vitrification covered wider HMDB categories, including lipids, heterocyclic compounds, benzenoids, oxygen organics, organic acids, alkaloid derivatives, nitrogen organics, phenylpropanoids and polyketides.

Detailed HMDB subclass classification (Figure 2D) showed forskolin-related metabolites included organooxygen compounds, prenol lipids, carboxylic acid derivatives, cinnamic acid derivatives, coumarins, fatty acyls, isoflavonoids, quinoline derivatives. Metabolites responding to vitrification involved fatty acyls, carboxylic acids, glycerophospholipids, organooxygen compounds, prenol lipids, steroids, benzenes, coumarins, flavonoids, indoles and other miscellaneous substances.

Disease- and immune-related pathways considered irrelevant to oocyte physiology were filtered out from the KEGG enrichment output for main-text interpretation. After filtering, KEGG enrichment analysis revealed significant enrichment of N-glycan biosynthesis (map00510, *p* < 0.05) in the forskolin-treated group. Vitrification triggered broader metabolic perturbation, with three significantly enriched metabolism-related pathways (*p* < 0.05): starch and sucrose metabolism (map00500), galactose metabolism (map00052), and glycine, serine and threonine metabolism (map00260). These KEGG enrichment results indicate that forskolin and vitrification perturb different metabolic signaling networks in oocytes, although the present metabolomic dataset alone cannot directly demonstrate that the two treatments reduce cellular lipid content via separate metabolic routes.

### 3.5. Lipid-Droplet Remodeling Prior to Vitrification Is Associated with Alleviated Mitochondrion-Mediated Early Apoptosis in Cryopreserved Oocytes

**Figure 3:**
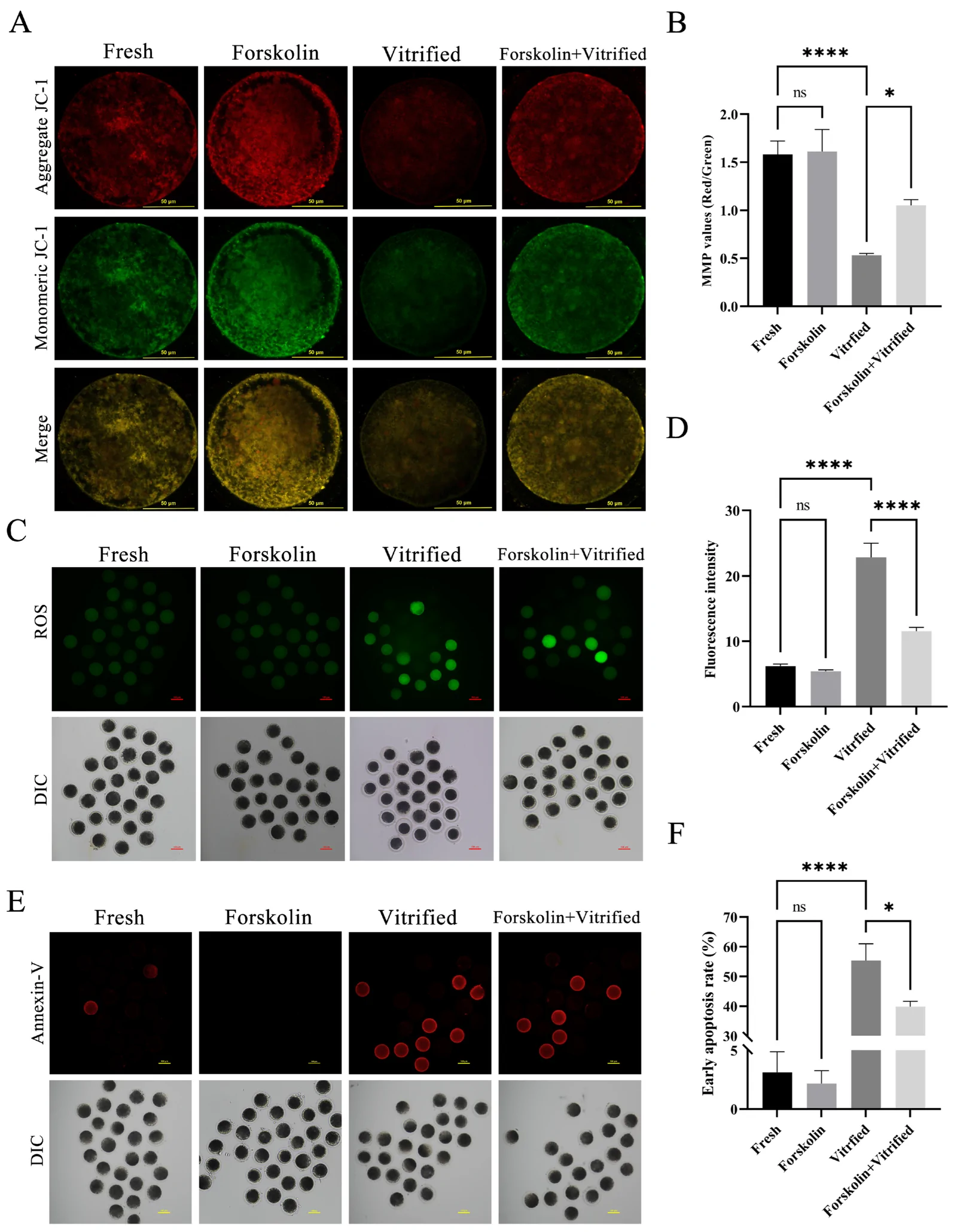
Determination of MMP, ROS level and early apoptosis in oocytes. (**A**) Determination of oocyte MMP. Scale bar = 50 μm. (**B**) Statistical analysis of MMP (*n* = 30). (**C**) Fluorescent images of oocytes after DCFH-DA staining. Scale bar = 100 μm. (**D**) Statistical analysis of oocyte ROS generation (*n* = 5). (**E**) Fluorescent images of oocyte Annexin-V staining. Scale bar = 100 μm. (**F**) Statistical analysis of oocyte early apoptosis (*n* = 5). * *p* < 0.05; **** *p* < 0.0001; “ns”, non-significant (*p* > 0.05).

We measured mitochondrial membrane potential (MMP), reactive oxygen species (ROS) levels and early apoptosis to evaluate mitochondrial function and redox homeostasis. Vitrification significantly decreased oocyte MMP (*p* < 0.01), suggesting vitrification triggers mitochondrial depolarization in porcine oocytes (Figure 3A,B). Mitochondrial depolarization commonly promotes massive ROS release. Accordingly, vitrified oocytes exhibited drastically elevated ROS levels (*p* < 0.01), indicating that vitrification induces severe oxidative stress (Figure 3C,D). Mitochondrial dysfunction further initiates intrinsic apoptosis. Consistently, vitrification markedly increased oocyte early apoptosis rate (*p* < 0.01) (Figure 3E,F).

Compared with vitrified oocytes alone, continuous forskolin supplementation before vitrification restored post-thaw MMP (*p* < 0.05), relieved oxidative stress by reducing ROS accumulation (*p* < 0.01), and ultimately lowered early apoptosis rates (*p* < 0.05).

### 3.6. Forskolin-Associated Lipid-Droplet Remodeling in Porcine Embryos

**Figure 4:**
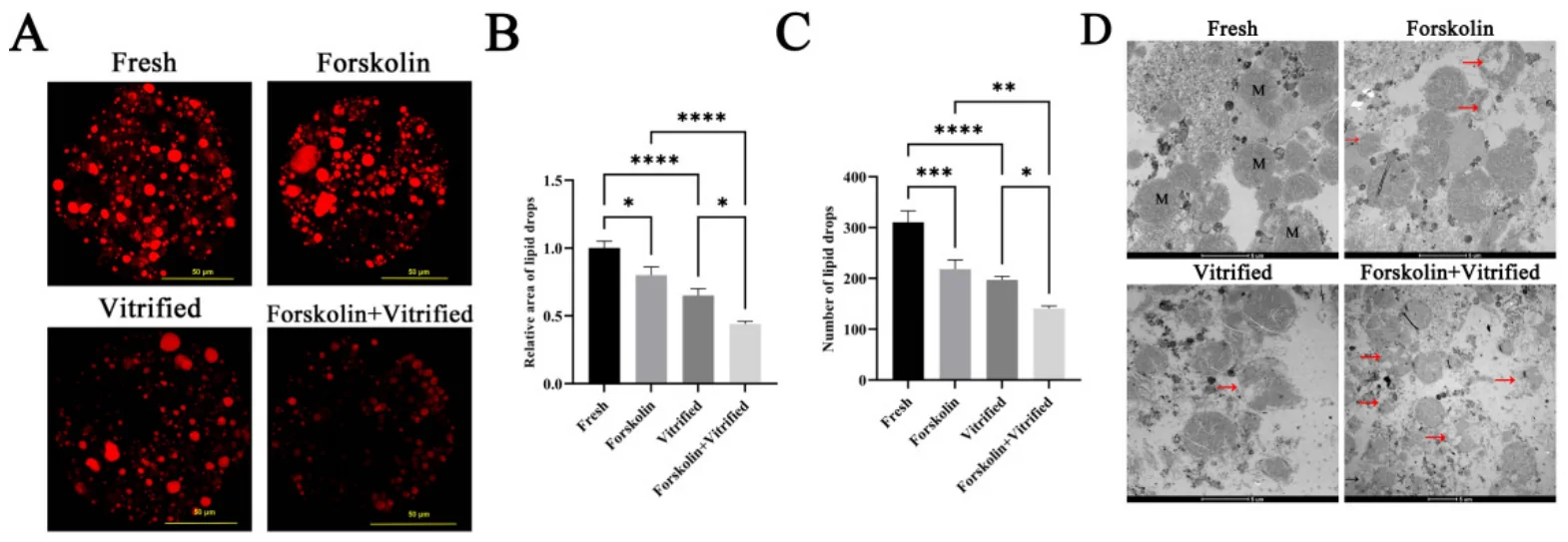
Fluorescent quantitation and ultrastructure of LDs in embryos. (**A**) Fluorescent images of LDs stained with Nile red in embryos. Scale bar = 50 μm. (**B**) Quantitative analysis of relative areas of LDs (*n* = 30). (**C**) Quantitative analysis of the numbers of LDs (*n* = 30). (**D**) Ultrastructural observation of embryos. M: heterogeneous LDs; red arrows: the degradation process of heterogeneous LDs; scale bar = 2 μm. * *p* < 0.05; ** *p* < 0.01; *** *p* < 0.001; **** *p* < 0.0001.

To verify whether this lipid-lowering strategy improves embryonic cryotolerance, we added 10 μM forskolin to both oocyte IVM medium and culture medium of parthenogenetic (PA) embryos. Embryonic LD changes presented consistent trends with oocytes. Either forskolin treatment or vitrification significantly reduced LD relative area (*p* < 0.05, *p* < 0.01) and LD number (both *p* < 0.01) in embryos (Figure 4A–C). Embryos receiving both forskolin and vitrification possessed the lowest LD number and relative area. TEM ultrastructural observation showed LD dissolution in all treated groups, marked with red arrows (Figure 4D).

### 3.7. Lipolysis Prior to Vitrification Alleviated Mitochondrion-Mediated Early Apoptosis in Cryopreserved Embryo

**Figure 5:**
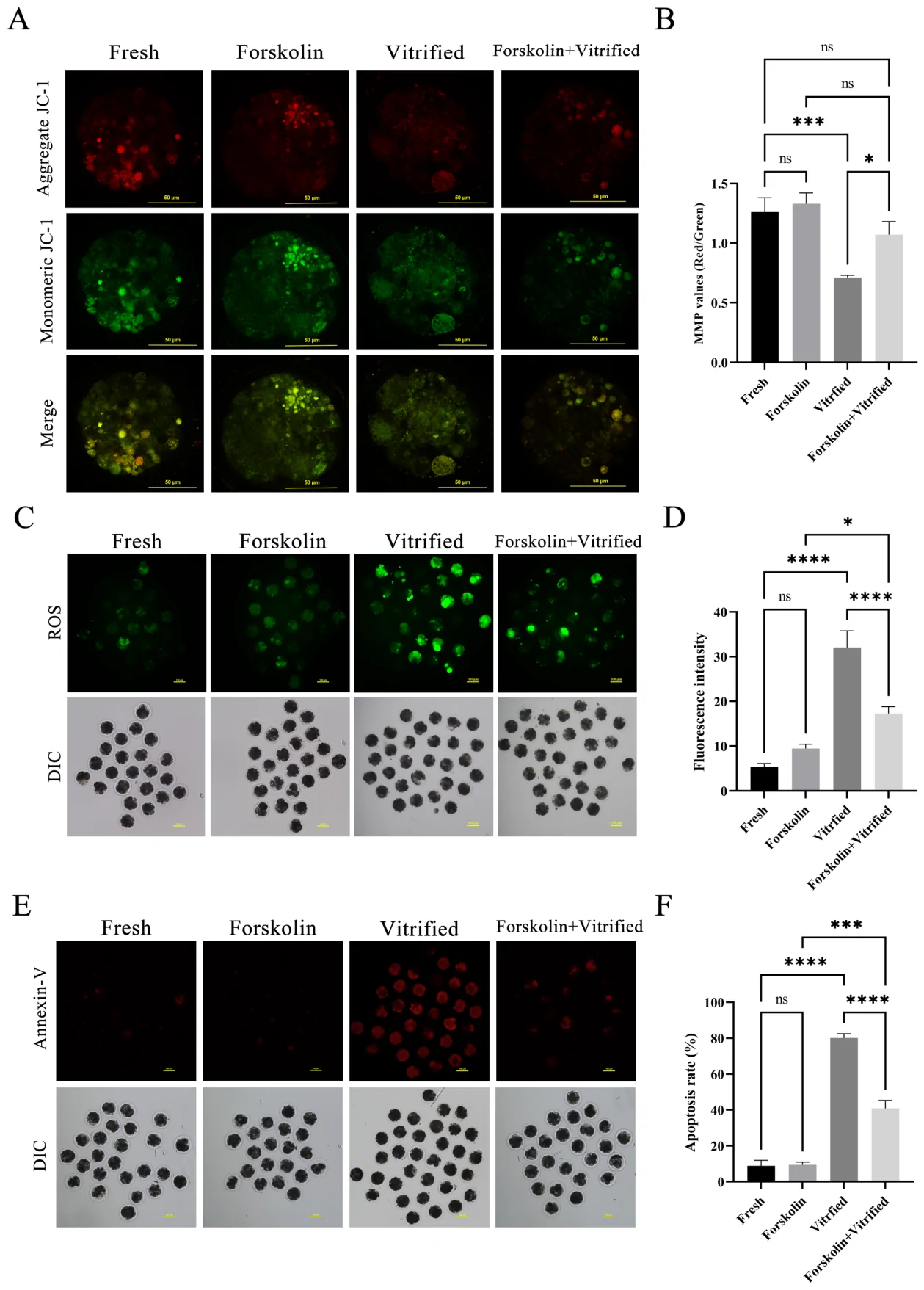
Determination of MMP, ROS level and early apoptosis in embryos. (**A**) Determination of embryo MMP. Scale bar = 50 μm. (**B**) Statistical analysis of MMP (*n* = 30). (**C**) Fluorescent images of embryos after DCFH-DA staining. Scale bar = 100 μm. (**D**) Statistical analysis of ROS levels (*n* = 5). (**E**) Fluorescent images of embryos with Annexin-V staining. Scale bar = 100 μm. (**F**) Statistical analysis of early apoptosis (*n* = 5). * *p* < 0.05; *** *p* < 0.001; **** *p* < 0.0001; “ns”, non-significant (*p* > 0.05).

We further detected whether sustained forskolin treatment maintained mitochondrial function and redox balance in vitrified embryos (Figure 5). Relative to fresh embryos, vitrification induced MMP decline, ROS elevation and higher apoptosis rates (all *p* < 0.01). Compared with vitrified embryos, embryos receiving continuous forskolin supplementation elevated MMP (*p* < 0.05), decreased ROS levels (*p* < 0.01), and inhibited early apoptosis (*p* < 0.01).

### 3.8. Lipid-Droplet Remodeling Is Associated with Improved Survival and Developmental Competence of Cryopreserved Oocytes and Parthenogenetic Embryos

**Figure 6:**
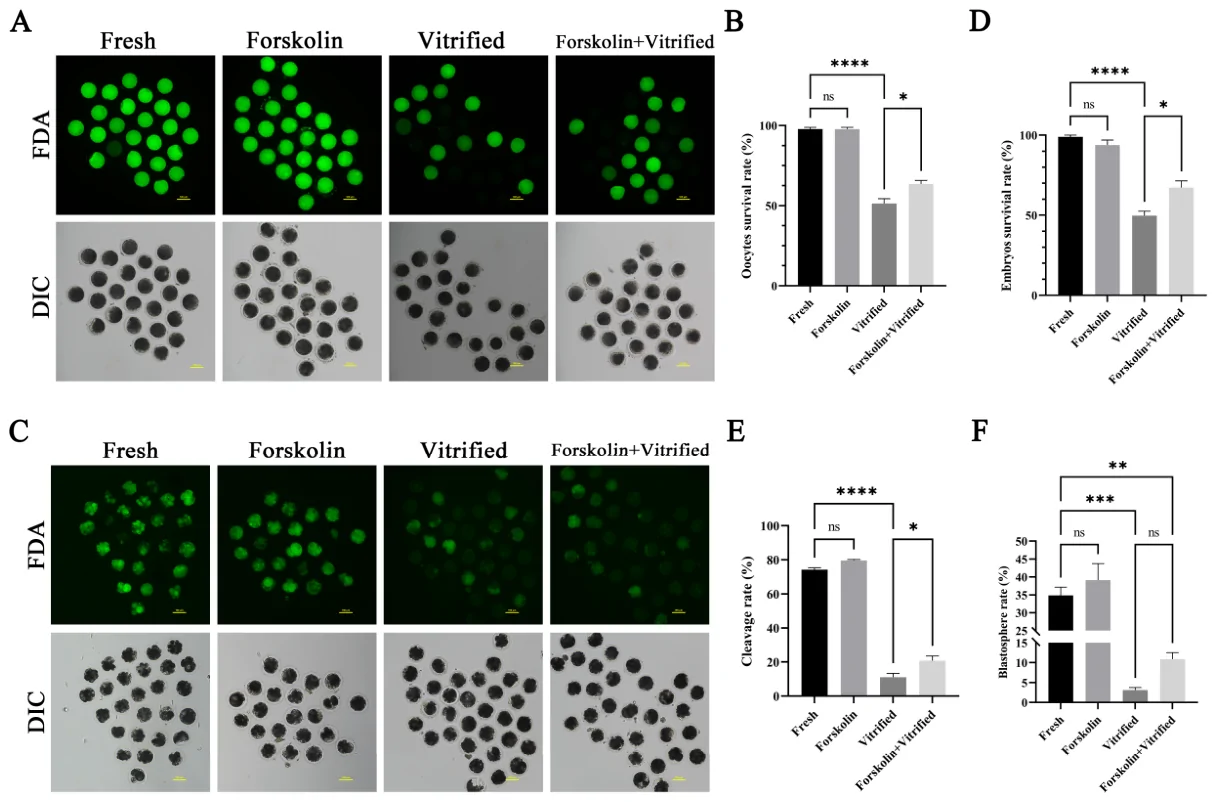
Determination of viability or embryonic developmental potential of oocytes and embryos. (**A**) Fluorescent images of oocytes with FDA staining. Scale bar =100 μm. (**B**) Statistical analysis of oocyte viability (*n* = 5). (**C**) Fluorescent images of embryos with FDA staining. Scale bar =100 μm. (**D**) Statistical analysis of embryo viability (*n* = 5). Ninety oocytes were involved in three technical replicates per group per biological replicate; 450 oocytes per group in total. (**E**) Statistical analysis of cleavage rate of PA oocytes (*n* = 5). (**F**) Statistical analysis of blastocyst rate of PA oocytes (*n* = 5). * *p* < 0.05; ** *p* < 0.01; *** *p* < 0.001; **** *p* < 0.0001; “ns”, non-significant (*p* > 0.05). Total oocyte numbers per experimental group was 750 across five biological replicates.

Survival (*n* = 5, 450 oocytes per group in total) and developmental competence (*n* = 5, 750 oocytes per group in total) were evaluated across five independent biological replicates. We detected post-thaw survival and developmental capacity to confirm the protective effect of forskolin-mediated lipid regulation against cryodamage (Figure 6). Vitrification significantly reduced survival rates of both oocytes and embryos (both *p* < 0.01). Compared with vitrified groups, continuous forskolin supplementation before vitrification significantly increased post-warming survival of oocytes and embryos (both *p* < 0.05).

For parthenogenetic developmental competence, vitrification significantly reduced both cleavage and blastocyst formation rates of oocytes (both *p* < 0.01; Figure 6E,F). Continuous forskolin supplementation markedly improved cleavage rates (*p* < 0.05). Although the average blastocyst rate numerically rose from 3.11% to 10.87% in vitrified oocytes following forskolin treatment, this difference was non-significant (*p* > 0.05; Figure 6F).

## 4. Discussion

**Figure 7:**
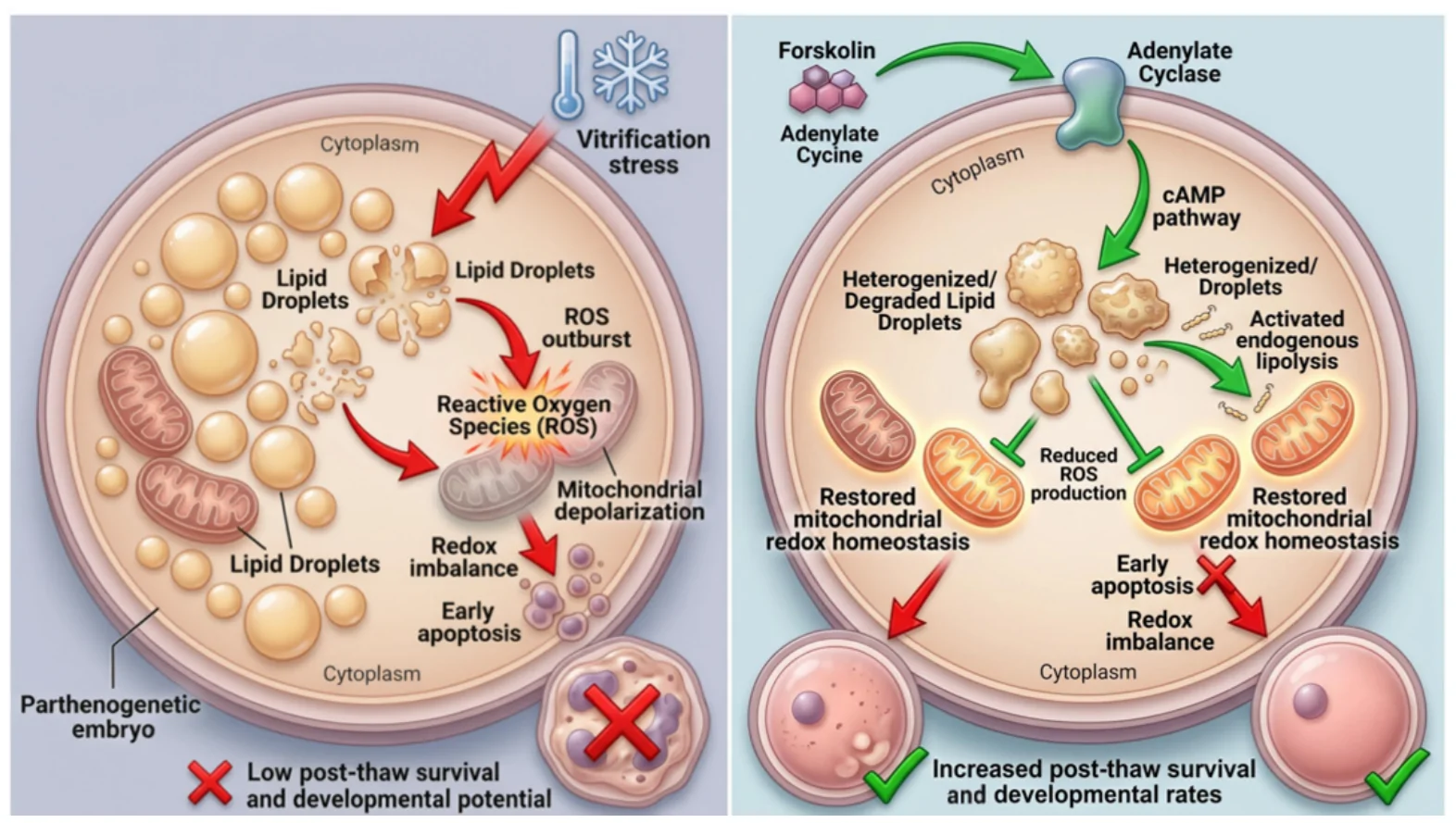
Schematic overview of the potential regulatory pathways involved in improving cryotolerance of vitrified porcine oocytes and embryos. Red arrows indicate adverse, damaging biological processes; green arrows represent protective signaling and beneficial cellular events. Red cross marks denote failed cell survival or inhibited biological processes; green check marks indicate improved cell survival and developmental competence.

Although viable offspring can be obtained from vitrified porcine oocytes in existing studies [9], the overall efficiency of porcine oocyte cryopreservation remains unsatisfactorily low. This technical bottleneck severely restricts the large-scale application of oocyte cryopreservation for the protection of precious indigenous pig genetic resources, and also limits the practical promotion of in vitro embryo production and embryo transfer in modern pig breeding systems. Given the indispensable value of embryo cryopreservation for long-term germplasm preservation and the intrinsically weak cryotolerance of porcine oocytes, it is urgent to optimize current cryopreservation protocols and elucidate the key regulatory mechanisms governing oocyte and embryo cryoprotection. The overall regulatory mechanism revealed in this study is illustrated in Figure 7.

Traditional embryo delipidation primarily relies on high-speed centrifugation combined with micromanipulation, which redistributes cytoplasmic lipid droplets and moderately improves the cryoresistance of porcine embryos [25]. Nevertheless, such physical invasive operations inevitably cause mechanical damage to intracellular structures. Moreover, these manipulative procedures increase the risk of pathogen contamination, thereby compromising oocyte viability and subsequent embryonic developmental competence [26]. Collectively, these inherent limitations of physical delipidation further emphasize the necessity and technical superiority of developing non-invasive metabolic lipid regulation strategies for germplasm cryopreservation.

Beyond livestock germplasm conservation, the present findings also provide broad mechanistic insights into mammalian reproductive cryobiology. The lipid-rich and cryosensitive characteristics of porcine oocytes closely mimic the cryopathological features of human oocytes, rendering the porcine model highly valuable for translational cryobiology research. In addition, cAMP-mediated lipid remodeling and mitochondrial redox homeostasis represent conserved regulatory pathways in mammalian germ cells. Accordingly, our exploration of forskolin-based metabolic intervention not only optimizes cryopreservation protocols for porcine germplasm but also deepens the understanding of lipid-dependent cryoinjury mechanisms, providing a feasible theoretical reference for improving oocyte cryotolerance and optimizing assisted reproductive technology across mammalian species.

In the present study, we first verified that forskolin supplementation during IVM exerted no adverse effects on granulosa cell growth and oocyte maturation. Although forskolin treatment resulted in a slight numerical increase in the rate of oocyte nuclear maturation, this difference did not reach statistical significance (*p* > 0.05), which is partially consistent with previous reports regarding the positive regulatory effect of forskolin on oocyte developmental competence [27]. This beneficial effect may be attributed to forskolin’s function as an adenylate cyclase agonist that modulates cAMP-related physiological processes. The stable and high maturation rate of forskolin-treated oocytes supports the feasibility of this non-invasive lipid-remodeling strategy for pre-vitrification optimization in porcine oocytes.

Porcine oocytes contain two typical types of LDs: homogeneous and heterogeneous LDs. Dynamic transformation between these two LD subtypes naturally occurs during oocyte development [28]. Severe low-temperature stress further triggers irreversible lipid phase transition of LDs in porcine oocytes during cryopreservation [29]. Our TEM ultrastructural observations indicate that vitrification induces severe structural destruction of intracellular LDs. After vitrification and warming, most homogeneous LDs transformed into heterogeneous LDs. Large-sized LDs decomposed into scattered small lipid particles, and partial LDs were completely dissolved, leaving only vacuolar structures in the cytoplasm.

Notably, forskolin treatment also induced gradual LD degradation and heterogenization in oocytes and embryos. We further focused on two core scientific questions: whether forskolin-mediated non-invasive lipolysis relies on metabolic pathways distinct from vitrification-induced lipid loss, and whether this metabolic regulation effectively alleviates cryogenic oxidative damage.

Intracellular LDs serve as critical energy reservoirs in oocytes and embryos. They sustain multiple essential biological functions, including meiotic arrest maintenance and triglyceride lipolysis via modulating cAMP signaling [21]. Compared with cattle, sheep and other livestock species, porcine oocytes accumulate excessive glycerolipids and fatty acids, resulting in unique lipid-rich cellular characteristics [30]. Such species-specific lipid metabolic features render porcine germ cells extremely vulnerable to cryoinjury, which can be comprehensively reflected by metabolomic profiling.

A recent study reported that vitrification exerted only moderate or minor effects on the transcriptome of in vivo-derived embryos, but significantly reshaped their metabolic profiles [31]. Our study complements the metabolic characteristics of vitrified porcine oocytes. We systematically characterized metabolomic alterations during oocyte cryopreservation and further clarified the differential metabolic mechanisms of LD reduction induced by pharmacological lipolysis versus cryogenic stress.

On this basis, we further explored the metabolic and physiological mechanisms responsible for the improved post-thaw survival of forskolin-pretreated oocytes and embryos. Our metabolomic comparison between single-treatment groups suggests that forskolin exposure and vitrification reduce intracellular lipid content via distinct metabolic signatures, which may contribute to divergent biological outcomes for oocytes. Pharmacological lipolysis-related remodeling may help prevent vitrification-induced cryodamage, whereas cryostress-mediated lipid loss exacerbates cellular dysfunction.

Mitochondria are core organelles governing energy metabolism and cellular redox homeostasis, and they are highly susceptible to low-temperature injury during cryopreservation [32]. Mitochondrial structural and functional stability directly determines the developmental potential of cryopreserved oocytes and early embryos [33]. Mitochondria are the primary source of intracellular ROS. Mitochondrial depolarization disrupts redox balance and triggers excessive ROS overproduction [34,35]. Our previous studies confirmed that vitrification-warming destroys mitochondrial crista structure, leading to mitochondrial dysfunction [36]. Damaged mitochondria further initiate oxidative stress and activate the mitochondrial-dependent early apoptosis pathway in porcine germ cells [37].

Apoptosis is the predominant form of programmed cell death responsible for cryopreservation failure and impaired embryonic developmental competence [38,39]. Accumulating evidence indicates that vitrification specifically induces apoptotic cascades in mature MII-stage porcine oocytes, rather than GV-stage oocytes [40]. Therefore, post-warming early apoptosis is a key restrictive factor for the survival of mature vitrified oocytes.

In this study, we systematically illustrated the protective mechanism of forskolin-mediated non-invasive lipolysis from the perspective of mitochondrial redox homeostasis. Forskolin preconditioning stabilizes mitochondrial function, suppresses vitrification-induced ROS burst and oxidative stress, and inhibits mitochondrial-dependent early apoptosis. Ultimately, this metabolic optimization significantly improves the post-thaw viability and developmental capacity of porcine oocytes and embryos. Collectively, our findings suggest that forskolin-associated lipid-droplet remodeling and vitrification-triggered lipid loss may arise from distinct regulatory networks, potentially leading to divergent cryopreservation outcomes in porcine germ cells. Direct biochemical evidence for lipolytic activation remains absent in the present dataset.

Several important limitations need to be acknowledged in this study. First, lipid-droplet ultrastructural changes and metabolomic data cannot provide definitive proof of lipolytic pathway activation, as biochemical assays for lipolytic products (free fatty acids, glycerol, and triglyceride hydrolysis) were not performed; LD heterogenization observed via TEM only suggests lipolysis-associated remodeling. Second, forskolin is a broad-spectrum adenylate-cyclase activator with diverse cAMP-dependent cellular effects. Thus, the improved mitochondrial membrane potential, reduced ROS levels and suppressed early apoptosis cannot be exclusively attributed to lipid-droplet reduction, and lipid depletion cannot be concluded as the principal protective mechanism. Other cAMP-dependent pathways may independently contribute to enhanced oocyte cryotolerance. Third, only parthenogenetic embryos were evaluated for developmental competence; these results cannot fully recapitulate outcomes from fertilized embryos, limiting direct extrapolation to commercial pig breeding or clinical assisted-reproduction applications. Fourth, the Forskolin + Vitrified group was not included in untargeted metabolomic profiling; therefore, our dataset cannot test whether forskolin rescues vitrification-triggered metabolic perturbation. Fifth, although all samples received identical 12 h serum-free incubation prior to metabolomic sampling to reduce serum-derived contaminant bias, this prolonged incubation may remodel endogenous oocyte metabolism and represents a potential confounding factor. Additionally, forskolin exposure regimens differ between oocyte and embryo experiments: oocytes were exposed to forskolin only during 44 h IVM, whereas embryo cultures received continuous forskolin supplementation throughout oocyte maturation and subsequent embryo culture up to vitrification, which should be considered when comparing oocyte and embryo results. Future investigations should incorporate direct biochemical assays for lipolysis, add the Forskolin + Vitrified group for metabolomic profiling, and assess fertilized embryos to further validate our findings.

## 5. Conclusions

This study established a non-invasive lipid-remodeling strategy during in vitro culture that may efficiently improve the cryotolerance of porcine oocytes and embryos. This continuous-exposure metabolic intervention approach provides a feasible and generalized research scheme to optimize porcine germplasm cryopreservation, with promising research value for the long-term preservation of rare and endangered pig genetic resources. Notably, our results cannot be directly translated to commercial breeding or human assisted reproduction practice. Furthermore, the present work offers novel insights into the procedural optimization of pig germplasm cryopreservation, especially regarding the effective treatment window prior to cryopreservation. Future studies may optimize forskolin concentration and intervention duration, or combine multiple lipid-regulating reagents to achieve further improved cryoprotective effects, which holds great potential for refining cryopreservation protocols for livestock germplasm.

## Data Availability

Data are contained within the article and Supplementary Materials.

## Supplementary Materials

The following supporting information can be downloaded at: https://www.mdpi.com/article/10.3390/cells15201909/s1, Table S1: Untargeted metabolomic dataset of porcine oocytes in positive and negative ionization modes. The table contains two worksheets: “pos_measurement” (5635 features detected in positive ionization mode) and “neg_measurement” (2881 features detected in negative ionization mode). Columns include metabolite feature information (feature ID, metabolite annotation, *m*/*z*, retention time, ionization mode, adducts, molecular formula, fragmentation score, theoretical fragmentation, mass error (ppm), KEGG compound ID, library ID, and CAS ID), followed by normalized peak intensities of six biological replicates for each of the three experimental groups (Fresh, Forskolin, and Vitrified) and three pooled quality control (QC) samples.
